# Cellulose processing using ionic liquids: An analysis of patents and technological trends

**DOI:** 10.1016/j.heliyon.2024.e39590

**Published:** 2024-10-18

**Authors:** Suellen Rocha Vieira, Jania Betânia Alves da Silva, Luiggi Cavalcanti Pessôa, Renata Quartieri Nascimento, Karina Lizzeth Pedraza Galván, Carolina Oliveira de Souza, Lucas Guimarães Cardoso, Jamille Santos Santana, Denilson de Jesus Assis

**Affiliations:** a Graduate Program in Chemical Engineering (PPEQ), Polytechnic School, Federal University of Bahia (UFBA), 40210-630, Salvador, Bahia, Brazil; b Center for Exact and Technological Sciences, Collegiate of Mechanical Engineering, Federal University of Recôncavo of Bahia (UFRB), 44380-000, Cruz das Almas, Bahia, Brazil; c Graduate Program in Biotechnology-Northeast Biotechnology Network (RENORBIO), Federal University of Bahia (UFBA), 40231-300, Salvador, Bahia, Brazil; d Graduate Program in Food Science (PGAli)–College of Pharmacy, Federal University of Bahia (UFBA), 40110-100, Salvador, Bahia, Brazil; e Department of Bromatological Analysis, College of Pharmacy, Federal University of Bahia (UFBA), 40110-100, Salvador, Bahia, Brazil; f Department of Engineering, University Salvador (UNIFACS), 41820-021, Salvador, Bahia, Brazil

**Keywords:** Cellulose, Dissolution, Ionic liquids, Mathematical model, Patents

## Abstract

The production of cellulose derivatives using ionic liquid (IL) as solvents and catalysts has become prominent over the last few years, since the process eliminates the use of toxic substances. This study aimed to map and understand the trends in cellulose processing using ILs by a patent analytic approach and technology life cycle modeling. The documents were searched on the Espacenet® and Orbit® platforms.The majority of innovations have come from companies based in developed countries. The data fitted to the sigmoid BiDoseResp model and the life cycle S-curve showed a market in an early stage of maturity. This mapping brings information that subsidizes decision-making regarding investments, research, and innovations aimed at IL-mediated cellulose treatment. Potential markets mostly use ILs of the imidazolium family in polymer chemistry, machinery, and biotechnology technologies. However, medical and pharmaceutical technologies and microstructure and nanostructure applications are still emerging, fostering perspectives for innovation.

## Highlights

• Evolution and socioeconomic factors of IL-cellulose technology are critically reviewed.
• 50 % of patents focus on ILs for cellulose dissolution, showcasing their eco-efficiency.
• Imidazolium ILs dominate cellulose tech, driving advancements in multiple fields.
• Life cycle modeling shows IL-cellulose tech nearing early maturity, signaling readiness.
• Emerging IL applications include health, environment, and nanotechnology.

## Graphical abstract

**Figure:**
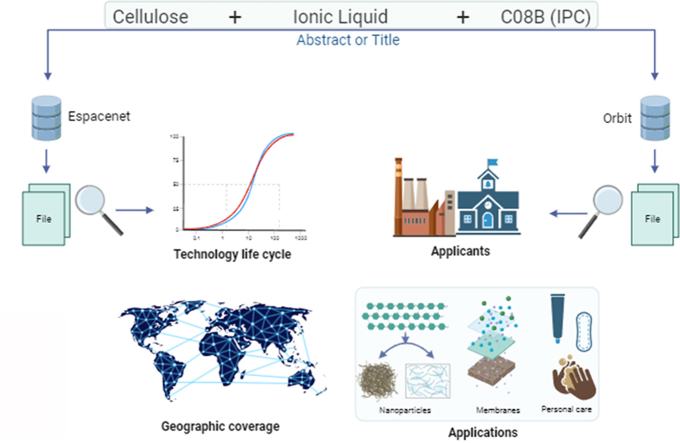

## 1 Introduction

Cellulose is considered the most abundant renewable polymer on earth. It is organized as microfibers linked together to form cellulose fibers, which in turn have crystalline and amorphous regions. The amorphous regions are susceptible to acid attack and can be removed under controlled conditions, leaving the crystalline regions intact [1,2].

The cellulose sector is very representative for the world economy due to the large revenue generated, high investment, and impact on several economic sectors [3]. The industries are technologically developed and use industrial facilities with large production capacity. In 2019, the cellulose market was valued at USD 211.68 billion, with estimates of 2.9 % annual growth rate from 2020 to 2026. Thus, the projected value for 2026 is USD 238.99 billion [4]. Factors justifying this growth include the increased paper production worldwide, the growing demand for forest-based textile fibers, and the demand from food, beverage, and pharmaceutical industries [4].

Cellulose is classified into different types according to the organization of its fibers. Long-fiber cellulose (2–5 mm in length), extracted mainly from coniferous trees such as pine, provides greater resistance to products and is widely used in the production of paper and packaging that require durability. Short-fiber cellulose (0.5–2 mm in length), extracted from eucalyptus, offers less resistance, but provides greater softness and excellent absorption. Pine stands out for its high capacity for absorption and retention of liquids [5,6]. In addition to the organization of the fibers, natural cellulose has four distinct polymorphs: types I, II, III and IV. Polymorphs I and II have a crystalline structure, while types III and IV are amorphous forms, obtained through various chemical reactions [1]. The structure of cellulose, combined with its ability to be modified through different chemical processes and physical, opens up countless possibilities for the development of new products in various sectors.

Cellulose dissolution is an important process in the production of fibers, films, and other derivatives. This process involves the disintegration of its crystal structure and water–polysaccharide bond formation [7,8]. Most methods involve high temperature, pressure, and solvent quantity, as well as heating cycles and vigorous stirring. Cellulose use is restricted due to its limited dissolution in the most common solvents, being soluble in toxic organic solvents [7] and sulfuric acid, which is the most used substance for this process [9]. An ideal solvent to dissolve cellulose, which include ionic liquid (IL), should have high solubility, fast dissolution, and low toxicity [8,10,11].

ILs are a group of salts that can replace volatile organic acids due to their low volatility, melting point, and toxicity as well as easy handling and storage. In the last three decades, ILs have attracted attention due to their unique physical and chemical properties, proving to be promising for various industrial applications [[12], [13], [14]]. Since they dissolve several polar and non-polar compounds, they are considered promising solvents for several applications, such as cellulose transformation to obtain derivative products, lignocellulosic biomass pretreatment, cellulose acylation and silylation, and cellulose ester and nanocrystal production [15].

ILs have a promising market potential. In 2020, the global market exceeded USD 1.40 billion and is likely to grow at 18.4 % annual rate, with the projected market being USD 4.69 billion by 2027 [16]. This growth is driven by factors such as the environmental impact, stimulating the demand for IL use as solvents and catalysts, and the innovative research developed for their application [16].

Although the application of ILs in cellulose has been thoroughly reported, particularly because ILs are economically and environmentally viable, few studies have focused on the origin, evolution, socioeconomic context, and life cycle of this technology. Therefore, patents are important data sources worldwide, since they deal with technological innovations of market interest.

Technology foresight is fundamental in decision-making regarding research, development, and innovation, which are fundamental components stimulating the organization of innovation systems in the business and academic spheres. Industry and technology mapping from patent databases increases knowledge on the current, local, and global status of a technology [17,18], which are important to assess not only competitiveness, but also legal and commercial strengths [18]. Accordingly, this study aimed to map and learn the technological trends related to cellulose treatment with ILs using a patent analytical approach and technology life cycle modeling, also highlighting the main holders of this technology and its applicability in different sectors.

## 2 Methodology

Patent data were retrieved from the European Patent Office (EPO) bank (Espacenet®) (https://worldwide.espacenet.com/patent/search), which includes documents filed in more than 100 countries, and Orbit® (https://www.orbit.com/), a private patent search and analysis platform of the Questel company, with the same keyword and code combinations, to generate comprehensive visual analytical tools, particularly for searching the predominant technological domains and making graphs.

The search strategy comprised combinations of International Patent Classification (IPC) codes and keywords. The advanced search consisted of filling the “title or abstract” field with the keywords “cellulose” and “ionic liquid” to know the worldwide panorama of IL use in several sectors. The search was then refined using several combinations of the keyword “ionic liquid” and the codes C08 (organic macromolecules, preparation, and after treatment thereof) and C08B (polysaccharides and derivatives) in the “IPC” search field between 2000 and 2021.

The data were exported from the Espacenet® to the Microsoft Excel® and analyzed considering the indicators: IPC, filing year, inventors, applicants, country of origin, application area, and technological domains. The data in the abstracts helped to identify details of the innovations, such as IL composition, predominant product, and process application.

The cumulative number of patents was calculated for the intervals between 2000 and 2021. The data were adjusted using OriginLab software (OriginPro 8.1) to different sigmoid growth models (Boltzmann, BoltzIV, DoseResp, BiDoseResp, Logistic, SGompertz). As stated by Marinakis (2012) [19] and Smil (2019) [20], technology diffusion is comprehensively accepted as tracing a sigmoidal curve that is similar to a biotic growth path. Therefore, those empirical growth models are useful for mimicking technology diffusion data.

The quality of the fit of the mathematical models was evaluated using Analysis of Variance (ANOVA) at a 5 % significance level, calculated using OriginLab software (OriginPro 8.1). The p-value was determined to assess the significance of the results and was considered statistically significant when p < 0.05. The F-test was applied to compare the variability explained by the models with the residual variability. The F-statistic was calculated as the ratio between the mean square of the model and the mean square of the error. The highest values of the coefficient of determination (R^2^) and F indicated the best-fitting and most predictive model [21]. Technology life cycle was interpreted based on the analysis of the S-curve of the technological phase characterization, as proposed by Cantú and Zapata [22].

## 3 Results and discussion

### 3.1 Data search and retrieval strategy

Table 1 shows the search strategies for patents using the keywords “cellulose” and “ionic liquid” and international codes to narrow the search. Therefore, the combination of keywords and C08B code was chosen due to the specificity of the content included and the number of documents found.

**Table 1:** Strategy for searching patents.

Ionic^a^ and liquid^a^	Cellulose	C08	C08B	Total (EPO)
X				>10,000
X	X			1287
X		X		3869
X			X	488
X	X	X		568
X	X		X	252^a^

C: chemistry and/or metallurgy; C08: organic macromolecular compounds, chemical preparation or working-up, after treatment thereof; C08B: polysaccharides, derivative; EPO: European Patent Office.

a Patents analyzed in this study.

The total data corresponded to 584 patents, but only 252 documents were available for analysis as the others were still in the 18-month confidentiality period. The same combination of words and code was applied to the Orbit®, with 277 documents being obtained.

### 3.2 Annual evolution

**Fig. 1:**
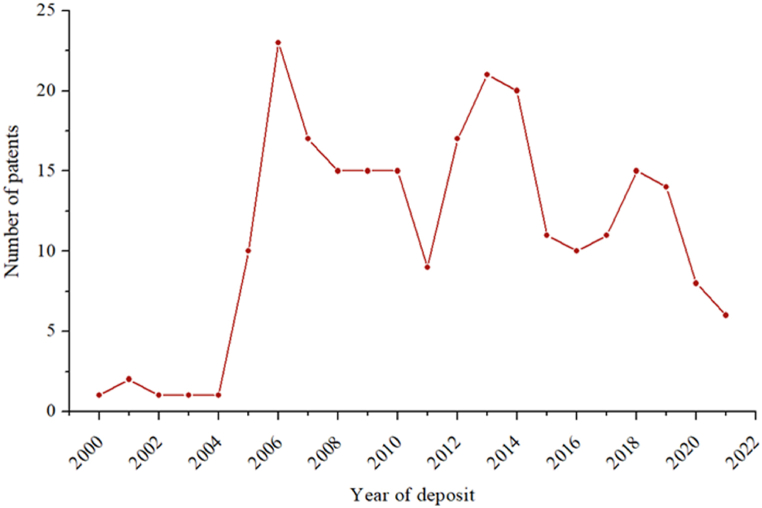
Annual evolution of patents (2000–2021).

Fig. 1 shows the annual evolution of patents filed in the last 21 years. Data behavior suggests that the growth of this technology peaked at 2006, with oscillations between 2006 and 2013. Subsequently, the number of patents decreased, which may be associated with the 18-month confidentiality period.

The first patent related to IL cellulose processing was filed by the chemical company British Celanese in the UK [23] in 1946, which reported improvements in organic cellulose ester production. The next application was made in 1952, also in the UK [24].

In 2006 (23 documents), 82 % documents corresponded to applications by the company BASF. Most patents reported methods for dissolving cellulose in IL [[25], [26], [27]], as well as the use of imidazolium family ILs [28], and quaternary ammonium [29] for carbohydrate dissolution, and polysaccharide (cellulose) silylation and acylation for application in textile, food, construction, and paint industries. This shows an increased interest in studying IL for dissolution, whose use gradually increased. According to the financial report, BASF spent EUR 631 million in 2006 in the plastics segment, which is 28.8 % higher than that spent in 2005, mostly to develop Ecoflex® (the company's first certified biodegradable compostable polymer) [30]. According to Speziali and Sinisterra [18], BASF produces various ILs that act as chemical inputs, carbohydrate solvents, and key components in efficient separation and rectification processes.

In 2005, the “Kyoto Protocol” was implemented to set targets for reducing the emission of harmful gases into the atmosphere and valuing chemical products produced from renewable resources. However, the treaty was signed in 1997 and several sustainable and renewable technologies have been developed since then [31,32]. Thus, in 2006, the interest of companies in the use of ILs increased from an economic, environmental, and social point of view.

In 2010, global cellulose export increased by 5 % compared to that in 2000. In 2010, the global largest cellulose producers were the USA (49 million tons, 26.5 %), China (22 million tons, 11.9 %), Canada (18 million tons), and Brazil (14 million tons) [33,34]. The USA is the largest consumer and Brazil's main trading partner, accounting for 21 % export in 2010 [34]. In the same year, China became the largest cellulose importer worldwide, accounting for 24 % import [33]. This increased cellulose production also increased the interest in research and, consequently, filing of patents related to cellulose and IL.

In 2013, the number of applications further increased, with 21 documents being filed and 80 % of them reporting innovations regarding polysaccharide derivative production, methods for producing cellulose esters and acetate, polymer treatments using IL, and lignocellulosic biomass production.

Among the patents filed in 2020 and 2021, more than 60 % intellectual property corresponds to universities and research institutes. This is due to the investment in the promotion of technology by the universities.

The most recent available patent document [35] reports an invention by the Xuzhou University of Technology, in China, which involves pretreating cellulose using the binary system 4-butyl-3-methylimidazolium hydrogen sulfate ([BMIM]HSO_4_) and ethanol (ET). The method consists of mixing [BMIM]HSO_4_ and ET solutions and adding the microcrystalline cellulose (MCC) with stirring and heating, to obtain a treated cellulose.

### 3.3 Technology life cycle

Table 2 presents the adjustment statistical data for the growth models. The cumulative patent data showed good adjustment to the tested models except BoltzIV (R^2^ > 0.99). Among the well-adjusted models significant for the F-test (p < 0.01), the BiDoseResp model showed the highest correspondence with the data (99.84 %) and the F-value (5010.33), showing that the mean squares of the model are larger than those of the residual, thus being more significant than the others.

**Table 2:** Cumulative number of documents adjusted to the sigmoid growth models.

Model	F-Value	P-Value	R^2^
Boltzmann	3070.08	<0.01	0.9954
BoltzIV	695.91	<0.01	0.9797
DoseResp	3070.08	<0.01	0.9954
BiDoseResp	5010.33	<0.01	0.9984
Logistic	3076.28	<0.01	0.9954
SGompertz	5797.28	<0.01	0.9967

Equation (1) presents the BiDoseResp model, which shows double sigmoid behavior, revealing the presence of two phases in the development of the prospected technology [36,37].

(1) *Y= A*_*1*_*+(A*_*2−*_*A*_*1*_*) × [p/(1 + 10^(t*_*1*_*−t)h*_*1*_*)+ (1−p)/(1 + 10^(t*_*2*_*−t)h*_*2*_*)]*

Here, Y is the cumulative number of patents, A_1_ is the asymptotic value at the bottom of the curve, A_2_ is the asymptotic value at the top of the curve, p is the ratio between the two segments, h_1_ is the slope coefficient of the first segment, h_2_ is the slope coefficient of the second segment, and t_1_ and t_2_ are the characteristic times of the two segments, respectively.

**Fig. 2:**
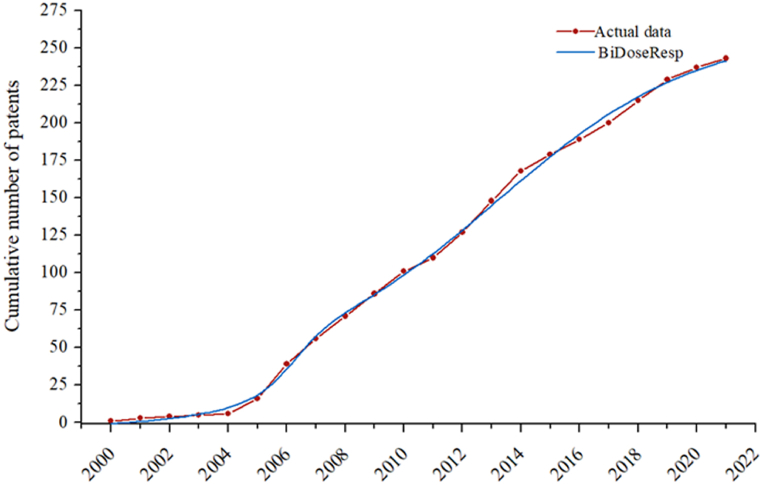
Technological trend of cellulose processing with ionic liquid (IL).

Fig. 2 shows the cumulative distribution of filed patents involving cellulose processing with IL and the sigmoid model (BiDoseResp) to identify the technology maturity stage.

The adjustment of the trajectory of the technological trend of IL cellulose processing to the BiDoseResp model (Fig. 2) developed mathematical data (Table 3) that helped identify the stages of technology development.

**Table 3:** Parameters of the BiDoseResp model for the technological trend of cellulose processing with IL.

	Variables							
	A_1_	A_2_	t_1_	t_2_	h_1_	h_2_	P	
Value	5.425	262.740	2006.210	2013.230	0.697	0.127	0.150	
Standard error	5.172	8.528	0.218	0.489	0.339	0.017	0.060	

The results showed an insufficient difference between the phases (segments) of the technology analyzed. The first phase started in 2000 and declined in 2007, while the second phase was ongoing. The ratio between segments was <0 (p = 0.150), indicating that the amplitude of the first curve segment is smaller than the amplitude of the second [38].

Table 3 shows t_1_ and t_2_ values, which indicate that the first and second technology phases reached state-of-the-art development stages in 2006 and 2013, respectively. These years mark the maximum development of a particular technique in inventive evolution (cutting-edge technology characteristic regions in sigmoidal curves) [36] and have the highest patent application rate (h). Thus, the interest in innovations involving IL-mediated cellulose processing showed the highest growth rate in the first phase (h_1_ = 0.697). In the second phase, the growth rate was five-fold lower (h_2_ = 0.193) than that in the first phase. This behavior is expected because an intense growth sector indicates a patent race to overcome obstacles before reaching the industrial commercialization phase, while a slow profile with a tendency to stability indicates sector maturity.

**Fig. 3:**
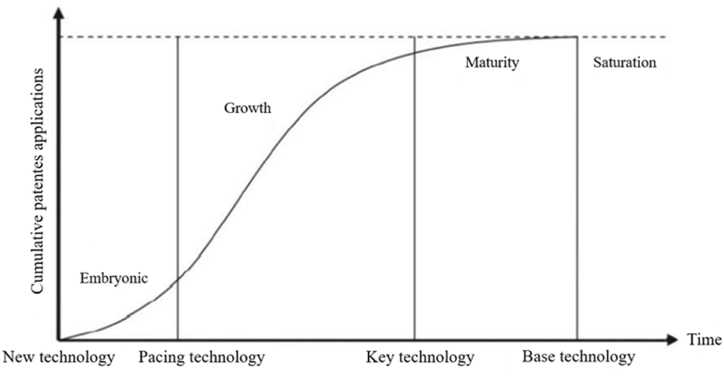
Typical technology life cycle S-curve. Source: Carvalho et al. [37].

The overlapping curves (Fig. 2, Fig. 3) indicate that the development of cellulose processing technologies using ILs is at an early stage of maturity, characterized by the tendency to become key technologies and integrate products or processes of highly competitive impact [37], such as energy, environment, biotechnology, and advanced materials.

In general, companies with capital strength and technical capacity influence the growth and stimulation phase, developing differentiated products to conquer the market [39].

### 3.4 Technology-holder countries

**Fig. 4:**
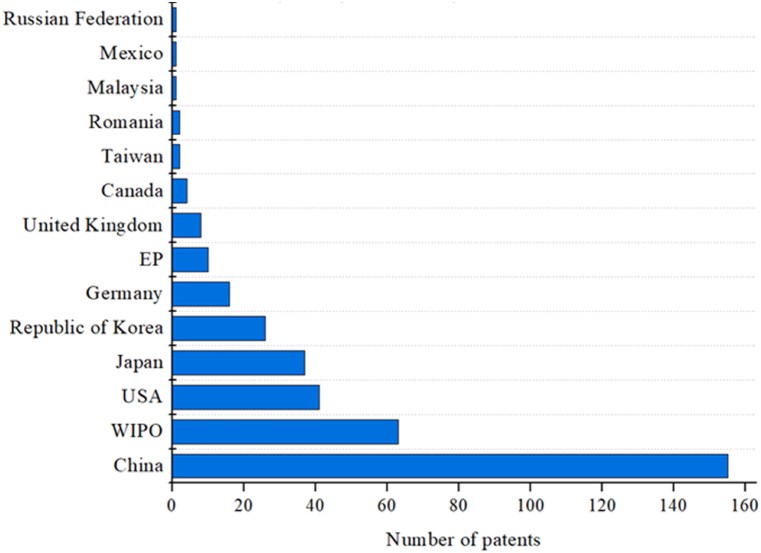
Number of patent applications by countries.

The analysis of the origin of patent applications shows that most technologies analyzed have been developed in the developed countries such as China and the United States of America (Fig. 4).

China (155 patents) is the main country holding IL cellulose processing technology, followed by the World Intellectual Property Organization (WIPO) (63), the United States of America (USA) (41), and Japan (37).

China's leadership in the race for technology patents can be attributed to several challenges the country faces, which have, in turn, driven significant investments in sustainable technologies. Its vast population and the resulting demand, combined with limitations in arable land and petroleum reserves, have spurred the development of biomass-focused alternatives. The Chinese government, together with its ministries, plays a critical role in mitigating the risks associated with innovation, adopting an entrepreneurial approach that promotes public-private collaboration [40]. This proactive stance toward innovation, particularly in green technologies, helps China sustain its global leadership in patent applications.

The first application filed in China was by CHINESE ACAD INST CHEMISTRY, in 2002 [41], which reported an IL containing unsaturated double bonds at room temperature and its application in dissolving cellulose. In general, the subsequent patents dealt with the cellulose transformation using ILs and mixed solvents, cellulose depolarization methods, film production, cellulose esters, cellulose carbamate, and lignocellulosic biomass processing.

The first patent document on cellulose processing with ILs in the USA was filed in 2005 [42] by the University of Alabama, presenting methods to prepare cellulose-based biocomposites using IL.

The USA is the world's largest and main cellulose consumer, followed by China, Japan, and Germany [43,44]. The high cellulose consumption by these countries may be justified by their tendency to develop clean technologies stimulated by environmental policies. The USA is the largest research and development (R&D) investor worldwide (USD 476.5 billion annually), followed by China (USD 370.6 billion), Japan (USD 170.5 billion), and Germany (USD 109.8 billion) [45].

The USA has been the global scientific and technological leader for decades. Multiple institutions contributing to the American science and technology system compete for allocated government resources, increasing system dynamism and efficiency. In 2009, they invested an extraordinary amount of money in R&D, stimulating economic growth after the 2008 crisis. In 2013, they invested approximately 2.8 % gross domestic product (GDP) in R&D [46].

In 2016, China increased its foreign investment in renewable energy by 60 %, to USD 32 billion that includes overseas investments in 11 new businesses worth more than USD 1 billion each. In 2015, China invested more than USD 100 billion in clean energy, which is more than double the US investment and a stimulus for job creation [37]. In addition, according to the Chinese National Bureau of Statistics, by 2020 China will have increased its R&D spending by 10.3 %, reaching USD 378 billion.

In 2023, a report by the Australian Strategic Policy Institute (ASPI) [47] revealed that China is outperforming the USA in 37 out of 44 crucial technological fields, including defense, space, robotics, energy, environment, biotechnology, artificial intelligence, advanced materials, and key quantum technology areas. The USA currently leads in areas such as high-performance computing, quantum computing, and vaccines. The report attributes part of Chinese potential to its scientific relevance, as the country hosts the top 10 research institutions in some of the 44 technologies, which generate nine-fold more high-impact studies than that produced by the USA. In addition, the country imports talent and knowledge through cooperation with researchers from the Five Eyes Intelligence Alliance countries (USA, UK, Canada, Australia, and New Zealand).

### 3.5 Technology holders

The global cellulose market used to be fragmented by small and large market participants offering the same product. Thus, with the growing demand for environment-friendly and biodegradable products, prominent industry players are investing in the development of new products to strengthen their expertise in cellulose materials and gain a competitive advantage. Some major cellulose manufacturers are the Shin-Etsu Chemical Co., Ltd., Eastman Chemical Company, Dupont De Nemours, Celanese Corporation, and Bracell. The main strategies adopted by these manufacturers include reverse integration, acquisition, production capacity expansion, and new product development [4].

ILs are suitable alternatives to organic solvents in cellulose processing for reducing waste generation, being environment-friendly, and presenting low toxicity [48]. It is a safer substitute for strong acids, with great market opportunities. Due to economic growth, the chemical sector is expected to increase the demand for ILs. The global participation of the IL industry is highly consolidated and includes several major companies such as BASF, Tokyo Chemical Industries, Merck Chemicals, IOILTECH GmbH, and Solvay. Major manufacturers are mainly engaged in strategic initiatives, such as mergers, acquisitions, and partnerships, to enhance their research capacity and develop new technologies to expand the scope of IL use [16].

**Fig. 5:**
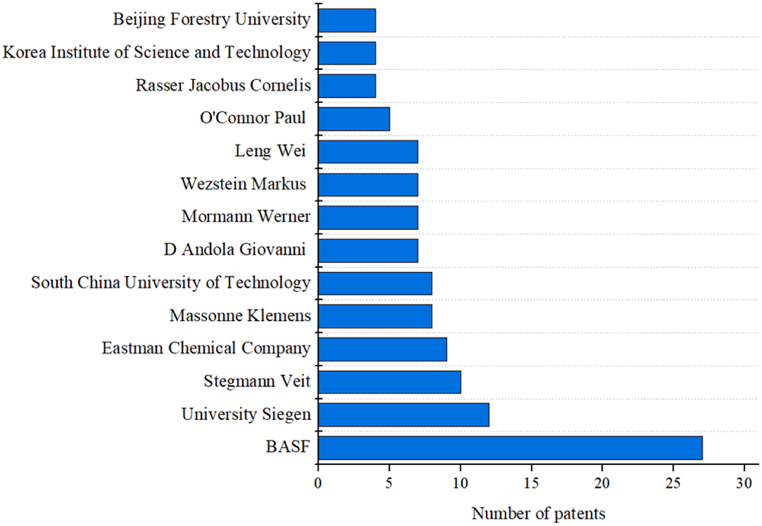
Applicants with the highest number of patent documents associated with cellulose transformation with ionic liquids.

Among the top technology applicants associated with cellulose transformation with ionic liquids (Fig. 5), companies are in first place, mostly chemical industries, followed by the universities that have a partnership with them. BASF has filed the most patents (27), followed by the University of Siegen (12), in Germany; the South University of Technology (10), in China; and the Eastman Chemical Company (9).

BASF is a German chemical company and the largest manufacturer of chemical products worldwide. Among the patents assigned to BASF, more than 44 % correspond to partnerships with the University of Siegen, a public research university located in Siegen, Germany (second largest patent applicant). The first partnership document was published in 2006. The University of Siegen focuses on cultural and social media research and basic and application-oriented research in sensor technology and nanoscience. Moreover, it supports research activities that capture future-oriented trends and new research areas. They present a competent area of nanotechnology and new materials that develops highly efficient materials and manufacturing processes that minimize resource consumption and maximize performance parameters that are ecologically sustainable [49].

BASF has the largest portfolio of patents and industrial applications and has been working in partnership with universities [50,51]. Biphasic acid scavenging utilizing ionic liquids (BASIL™), an industrial-scale process that won the 2004 Innovation for Growth award for trying to improve the unfavorable use of HCl, is a technology implemented by BASF in Germany (2002) [50]. The BASIL™ technology for eliminating acids, has been used by BASF for esterification, acylation, silylation, phosphorylation, sulfation, elimination, deprotonation, and acid removal in general in laboratory trials. This process is currently being considered for large-scale commercial use [52].

The patent documents evaluated report methods for dissolving cellulose in IL, polysaccharide (cellulose) silylation and acylation in IL for several applications, and cellulose acetal and polymer production. The company has concentrated on acetalization and acylation processes for sugars. In this case, IL is used as a solvent for the respective sugars. Moreover, some applications describe its use for cellulose dissolution considering the efficient solvent properties of ILs [18].

The Eastman Chemical Company is a global chemical company headquartered in Kingsport, Tennessee, USA, that specializes in manufacturing several advanced materials, additives, functional products, specialty chemicals, and fibers. Eastman invests in cellulose sugar to form plastics [53]. Buchanan Norma Lindsey and Buchanan Charles Michael are the main inventors at the Eastman Company, with nine filed patents. Most applications from this company focus on cellulose ester production and IL treatment [54,55].

The interest of universities in obtaining cellulose derivatives from ILs has increased from 2013, with most inventions focusing on cellulose dissolution methods, also justifying the peak in the yearly graph. The most cited cellulose derivatives obtained with ILs are hemicellulose and lignin, renewable lignocellulosic films, nanocrystalline film, cellulose membrane, cellulose aerogel, and cellulose nanocrystals. Methods for improving cellulose dissolution, mechanical performance in cellulosic material, and highly resistant nanocrystal production have been reported. Most patents filed this year have no partnerships with companies.

**Fig. 6:**
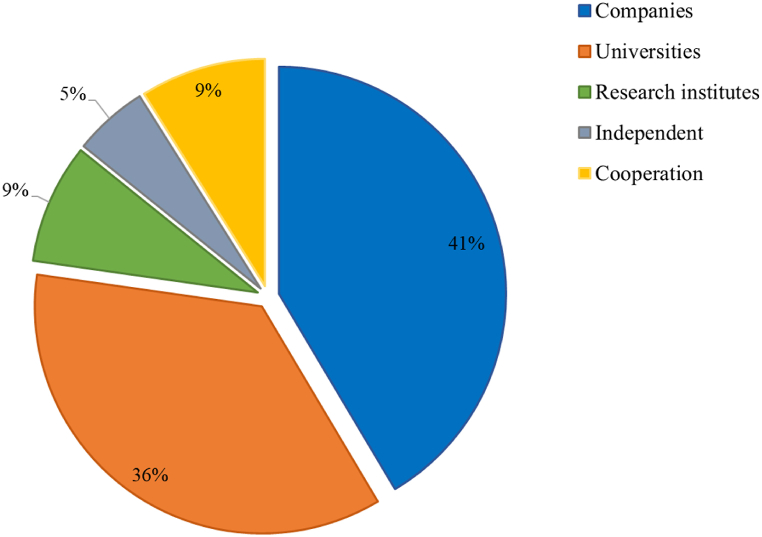
Distribution of patent documents by institutions.

Fig. 6 shows the distribution of patents by institutions. Companies have filed the largest number of patents (41 %), followed by universities (36 %), partnerships (9 %), research institutes (9 %), and independent inventors (5 %).

According to the BiDoseResp model, the distribution of sectors by technological cycle segments showed a significant contribution of companies (74.4 %) to the number of applications in the first period (2000–2007). This predominance shows the emerging interest in developing cellulose-derived processes and products with ecological and economic advantages. At this pace, most applicants are companies willing to take the R&D risk, since these technologies are still immature, with low competitive impact and low integration into products or processes [56,57]. Therefore, technologies in this segment were subject to fundamental scientific and technological problems that conditioned the development of marketable products. On the contrary, the second segment (2008–2021) indicated the most significant portion of the growth stage, with expressive participation of universities and research centers (59 %). Intuitively, we can attribute the protagonism of these institutions to the need for scientific support to increase the competitive impact and the transformation of emerging technologies into more suitable and safer technologies for the market.

### 3.6 Technology domains

**Fig. 7:**
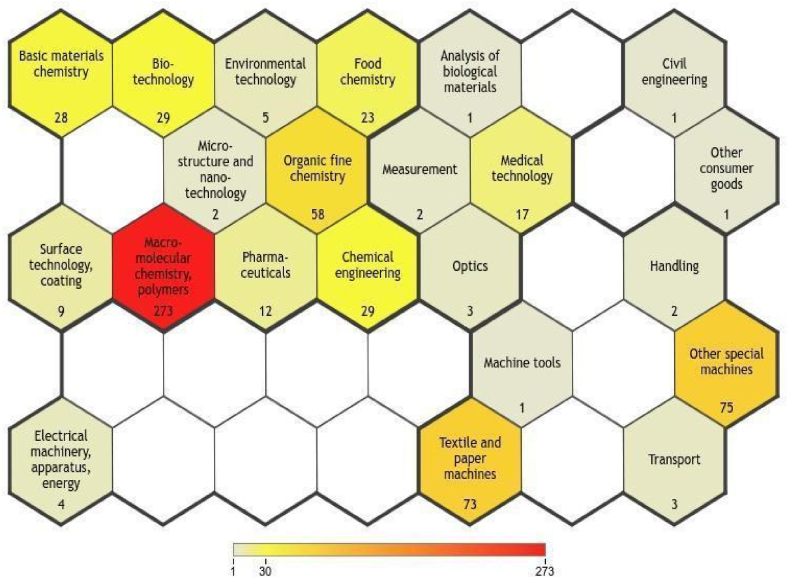
Technological domains associated with cellulose transformation with ionic liquids (ILs). Source: Orbit®.

Fig. 7 shows the diversity of economic sectors related to cellulose processing with IL. These data are based on IPC codes, grouped into 35 technology fields. Moreover, a patent document can be associated with multiple domains. Color intensity is associated with the number of documents in each technological domain.

The explored categories most associated with cellulose processing with IL are macromolecular polymer chemistry (273), textile and paper machinery (73), other types of machinery (75), organic chemistry (58), and biotechnology (29). All the documents related to the treatment of cellulose with ionic liquids fall within the technological domain of macromolecular polymer chemistry, and only one of these is associated with multiple segments. This is because ILs are substances that interact with polymers in various chemical and industrial contexts, as they are highly effective for dissolution and processing, enabling advances in the modification and application of cellulose-based materials. Additionally, the pretreatment of cellulosic biomass with ILs is an effective method for altering the supramolecular structure of polysaccharides and improving the efficiency of subsequent processing. It is primarily used for the dissolution or modification of biomass, facilitating a range of sustainable processes [58]. Medical technology (17) is an area that interfaces with cellulose processing with IL, which has not been explored well. The less represented categories identify other potential categories for patent applications such as pharmaceuticals (12), environmental technology (5), and microstructure and nanotechnology (2).

**Fig. 8:**
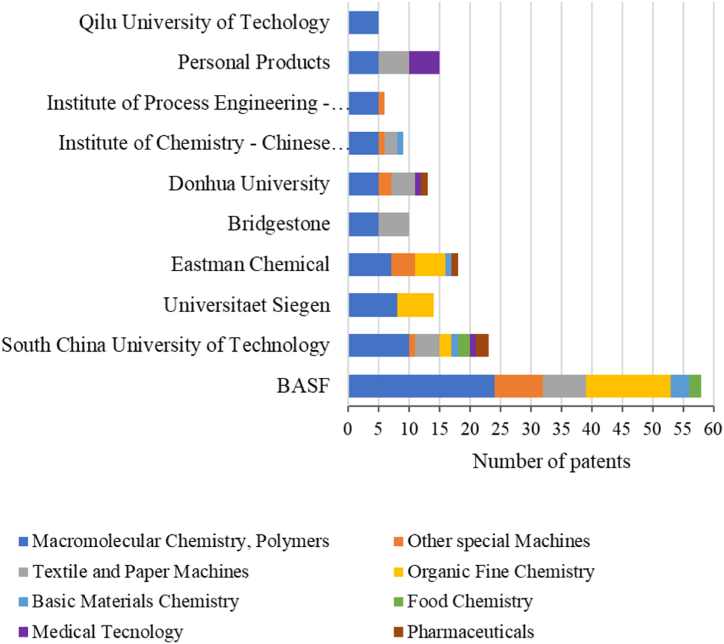
Technology domains associated with applicants with more than four filed patents. Source: Adapted from Orbit®.

Fig. 8 shows the technology domains associated with applicants with more than four filed patents. As shown in Fig. 8, macromolecular polymer chemistry represents the most associated category, and the applicants with more patents in this area are BASF (24), South University of Technology in China (10), the University of Siegen (8), and Eastman Chemical (7). All applicants with more than four filed patents show technological mastery in the macromolecular polymer chemistry category.

The South University of Technology is the applicant with the highest number of applications in different categories (8), followed by BASF (6), Eastman Chemical (5), and Donhua University (5). The University of Siegen, the second largest applicant of patents reporting cellulose processing using IL, focused only in the categories of macromolecular polymer chemistry and organic chemistry. The Eastman Chemical, the second largest applicant, focuses on the technology in the categories of macromolecular polymer chemistry (7), organic chemistry (5), other special machinery (4), basic materials chemistry (1), and pharmaceuticals (1).

The Personal Products Company filed five patents in the medical technology category. The Personal Products Company is a Johnson & Johnson subsidiary that manufactures innovative oral health, women's health, and health protection products. The company also works with 1 mg ARESTIN® (minocycline HCl) microspheres, which treats periodontal disease [59]. The patents GB1501239 [60], FR2308643 [61], DE2516380 [62], CH629228 [63], and OA05015 [64] are related to the production of cellulose graft copolymer as fibers with properties comparable to those of untreated cellulose and a high water- and aqueous liquid-absorbing capacity. They are therefore highly suitable as a component for absorbent materials such as dressings, menstrual hygiene materials, and diapers.

### 3.7 Area of technology application

**Fig. 9:**
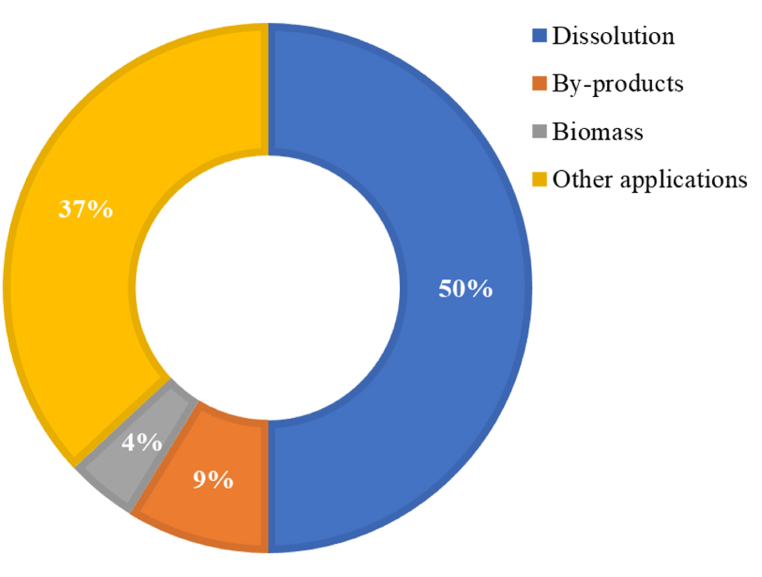
Patent distribution by application area.

Fig. 9 shows the distribution of patents by application area, with 50 % patents analyzed focusing on cellulose dissolution with IL, followed by IL use for cellulose by-product production (9 %), cellulose biomass production methods (4 %), and IL use for other applications (37 %).

ILs are efficient and environment-friendly solvents for dissolving polysaccharides [7,[11], [65]]. Cellulose processing using ILs is a green process that produces lignocellulosic materials, cellulose derivatives, and cellulose composites with various industrial applications [10] and CN104130332 [66].

**Fig. 10:**
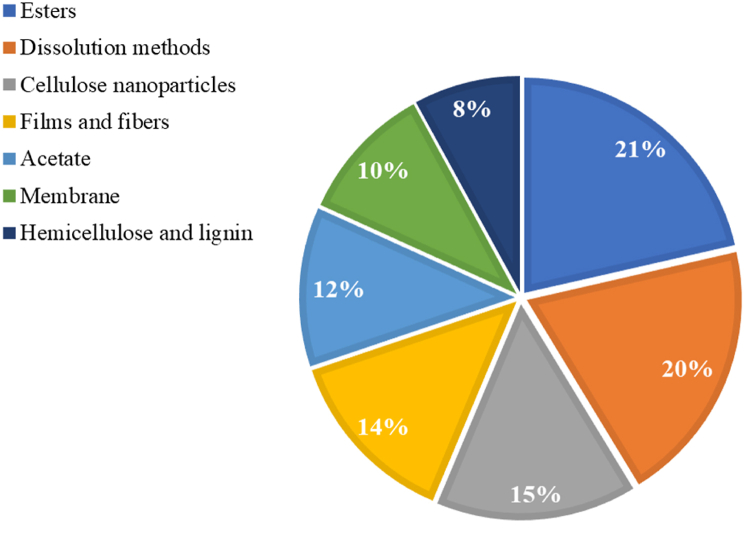
Patent distribution according to the application areas.

Fig. 10 shows the distribution of patents according to the application areas. Most patents retrieved report cellulose modification by adding functional groups such as esters and acetate (33 %), followed by innovations focused on cellulose dissolution methods (20 %) and nanocellulose production (15 %). Dissolving agents such as acetylation agents and liquids of the imidazolium family are used to produce these derivatives for application in plastic products and by-products and improve the mechanical properties.

ILs dissolve cellulose without derivatization at high concentrations. In addition, the liquid components (cation and anion) can be adjusted to improve the physicochemical properties affecting the performance of cellulose interactions [6], justifying their application in technologies involving this biopolymer.

Table 4 shows some ILs used in different cellulose processing processes for several applications.

**Table 4:** Ionic liquids (ILs) used in cellulose processing.

Document	Applicants	IL	Purpose
CN104130332 [6]	BEIJING CHEMICAL UNIV	1-butyl-3-methylimidazolium hydrogen sulfate	Cellulose ester
CN114044917 [35]	UNIV XUZHOU TECHNOLOGY	4-butyl-3-methylimidazolium hydrogen sulfate	Cellulose pretreatment
CN108774288 [67]	UNIV QILU TECHNOLOGY	4-butyl-3-methylimidazolium hydrogen sulfate	Cellulose nanocrystals
CN105622961 [68]	NAT UNIV DONG HWA	Tert-butyl acetate	Self-healing polysaccharide hydrogel
DE102006011076 [29]	BASF & UNIV SIEGEN	Quaternary ammonium	Films and fibers
CN109810295 [69]	UNIV QINGDAO SCIENCE & TECH	1-ethyl-3-methylimidazolium acetate	Cellulose film
CN114507299 [70]	KUNMING SCIENCE AND TECHNOLOGY UNIV	(3-chloro-2-hydroxypropyl) trimethyl ammonium chloride	Hemicellulose extraction
CN109280088 [71]	UNIV SHAANXI SCIENCE & TECH	1-(3-aminopropyl) imidazole	Antibacterial bacterial cellulose film
CN106800604 [72]	UNIV GUANGXI	1-butyl-3-methylimidazolium chloride	Nanocellulose preparation
CN107236048 [73]	UNIV QILU TECHNOLOGY	1-butyl-3-methylimidazolium chloride	Cellulose nanocrystal preparation
CN109762071 [74]	UNIV LUDONG	1-allyl-3-methylimidazolium chloride	Cellulose proline esters

Inventions reporting cellulose dissolution methods [[75], [76], [77], [78]] are major patents. Some technologies use cellulose dissolution methods with the IL imidazole [71], 1-butyl-3-methylimidazolium chloride [73,79], 1-allyl-3-methylimidazolium chloride [74], and quaternary ammonium chloride [29], which dissolve cellulose at low temperatures and in short time periods.

Swatloski et al. [80] reported that 1-butyl-3-methyl imidazole chloride can be used as a cellulose solvent. This is because cellulose can be slowly dissolved in ILs containing some anions at 100–110 °C to obtain different cellulose by-products.

Methods of producing cellulose films and/or fibers using IL have been reported in some documents [29,69,71,81,82], with most of them showing antibacterial and mechanical properties such as excellent tensile strength and elongation percentage.

ILs are used as solvents and catalysts in synthesizing nanocrystals [8]. Patents CN108774288 [67] and CN107236048 [73] report obtaining cellulose nanocrystals using [BMIM]HSO_4_ and 1-butyl-3-methylimidazolium chloride, respectively, followed by ultrasonication. The patents CN106674357 [83] and CN106800604 [72] report cellulose nanoparticle (CNP) production from MCC using EmimOAc IL and BMIM Cl, respectively.

CN109467608 [84] reports a method for preparing nanocrystalline cellulose from mulberry fiber, describing a pretreatment and a physicochemical treatment with IL that is being considered a green and efficient process with reduced preparation time. CN108774288 [67] reports a method to prepare cellulose nanocrystals using IL (BMIM HSO_4_) and MCC. Document CN106674357 [83], however, reports a method for preparing nanocellulose with EmimOAc IL and MCC, obtaining a product with high crystallinity and beneficial properties.

Cellulose dissolution can produce derivatives such as cellulose esters, which have industrial-scale applications in the paper, film, fiber, textile, polymer, coating, membrane, and composite industries [85]. The patents CN104130332 [67], CN104277121 [86], and KR101700106 [87] refer to the production process of these esters. CN107722127 [88] describes a method for preparing a reversible cellulose- and CO_2_-based IL for application after dissolution and reversible CO_2_ derivatization and cellulose activation.

WO2008100566 [89] also describes an invention process for cellulose ester production in carboxylated ILs. The method comprises dissolving cellulose in ILs and then placing it in contact with at least one acylation reagent. The cellulose esters produced may comprise ester groups originated from carboxylated IL and the acylation reagent. The synthesis of organic acid esters has been increasingly relevant due to demands in the chemical and pharmaceutical industries. Therefore, the traditional esterification method originates numerous variants by modifying the nature of the catalytic acid and/or the experimental conditions.

Patents classified as cellulose by-products include cellulose membrane production, electrolyte materials, functional materials, biocomposites, and cellulose biopolymers. Other patents describe the use of cellulose to produce ILs, cellulose acetals and cellulose for ion exchange, polymer treatment, and hemicellulose separation. Most of these patents describe preparation and dilution methods with ILs to obtain a green technology for processing and application.

DE102006042891 [25], DE102006030696 [90], DE102006042892 [91], and other patents refer to poly- or oligosaccharide acylation methods with ILs and the optional use of water and high temperatures. DE102006054213 [92] reported cellulose acetal production using IL. Barthel and Heinze [93] described reactions such as cellulose acylation and carbonylation in BMIM Cl without catalysts with short reaction period and at low concentration.

Several biomass pretreatment methods are used to remove lignin and hemicellulose, reduce crystallinity, and increase cellulose. This process includes the cleavage of ester groups and hydrogen bonds that release the degraded lignin, hemicelluloses, and cellulose [85].

Cellulosic biomass pretreatment and treatment methods (such as CN114044917 [35], WO2020234761 [94], and US2013252285 [95]) have been reported. WO2017024367 [96] reports a method for lignocellulosic biomass (sugarcane bagasse) pretreatment using protic ILs for sugar production via enzymatic hydrolysis in the production of second-generation ethanol and other fermentative process products. CN112064392 [97] reports a biomass pretreatment method and composition in which a high boiling point alcohol and an IL selectively dissolve the lignin, resulting in a gentle, efficient, and easy solvent recovery process.

Patent CN105017541 [98] reports a method to prepare a banana cellulose crystal and polylactic acid aerogel used in cellulose dissolution. CN105131317 [99] reports a method to prepare hydrogel membranes from bamboo shoot waste. The prepared hydrogel membranes have excellent mechanical properties, compact pores, and the potential application value of a permeable membrane that can be used in permeation, medicine, and cosmetic fields.

Cellulose preparation methods for biomedical applications were also noted. Patent CN103360550 [100] (A61L27/16; A61L27/60) reports a method to prepare a cellulose and polyisoprene graft copolymer using cellulose and isoprene as initial raw materials. Initially, cellulose is modified using an IL to prepare a cellulose macroinitiator. Then, a cellulose-graft-polyisoprene copolymer with excellent mechanical properties and high application value is produced, which can be used, for example, as artificial skin. CN105622961B [68] (A61L27/20; A61L27/52) reports a method for cellulose dissolution in IL to obtain a cellulose solution that is added to a chitosan solution to form a pH-responsive self-repairing polysaccharide hydrogel with self-healing performance. The preparation method is suitable to modify most polysaccharide derivatives, which have good prospects for application in tissue engineering and controlled drug release due to the good biocompatibility of cellulose and chitosan.

In the last 21 years, several technologies based on IL use in cellulose have been developed. However, innovations aimed at medical technology, pharmaceutical, and micro- and nanostructure applications are emerging. Therefore, obtaining cellulose nanoparticles by IL is a potential challenge for tissue engineering and, consequently, medicine.

## 4 Conclusions

This study mapped and modeled technological trends for using ILs in cellulose over the past 21 years, for identifying the unfilled gaps. The temporal evolution of innovations followed a sigmoid growth trend adjusted to the BiDoseResp mathematical model and the S-curve of technology life cycle, identifying the occurrence of two leading-edge development stages (2006 and 2013) and currently heads toward market maturity. This knowledge suggests a narrow gap for new technological contributions in the area, and provides a market landscape that, together with scientific evidence and technological advantages, can help competitive investors make decisions.

Most of the analyzed technologies use imidazolium family liquids for cellulose dissolution, modification, and fractionation, since they are categorized as “green solvents” owing to their physicochemical properties. Thus, the most explored domains include polymer chemistry, machinery, organic chemistry, and biotechnology. However, technologies for medical, pharmaceutical, and micro- and nanostructure applications are still emerging, fostering innovation perspectives.

The use of ionic liquids (ILs) for cellulose treatment has several future prospects. First, it can facilitate the development of sustainable materials, such as biodegradable packaging and eco-friendly textiles, by modifying the physical and chemical properties of cellulose. In biomedical applications, focusing on the production of cellulose nanoparticles can lead to innovations in tissue engineering and controlled drug delivery. Furthermore, the integration of ILs into sustainable production processes can optimize the conversion of lignocellulosic biomass into biofuels and bioproducts. While the scalability of these methods and cost reduction are crucial for the industrial adoption of ILs, interdisciplinary collaborations among chemistry, engineering, and biomedicine can accelerate innovations and increase energy efficiency, thereby contributing to the viability of processes using ILs without compromising sustainability.

## CRediT authorship contribution statement

**Suellen Rocha Vieira:** Writing – original draft, Methodology, Investigation, Formal analysis. **Jania Betânia Alves da Silva:** Writing – review & editing, Visualization, Conceptualization. **Luiggi Cavalcanti Pessôa:** Writing – original draft, Methodology, Data curation. **Renata Quartieri Nascimento:** Writing – review & editing, Methodology, Data curation. **Karina Lizzeth Pedraza Galván:** Writing – original draft, Conceptualization. **Carolina Oliveira de Souza:** Writing – review & editing, Formal analysis. **Lucas Guimarães Cardoso:** Methodology, Data curation. **Jamille Santos Santana:** Writing – review & editing, Visualization, Formal analysis. **Denilson de Jesus Assis:** Writing – review & editing, Supervision, Project administration, Investigation, Formal analysis.

## Declaration of competing interest

The authors declare that they have no known competing financial interests or personal relationships that could have appeared to influence the work reported in this paper.
